# Polymeric Microsphere Formulation for Colon Targeted Delivery of 5-Fluorouracil Using Biocompatible Natural Gum Katira

**DOI:** 10.31557/APJCP.2019.20.7.2181

**Published:** 2019

**Authors:** Saumen Karan, Hira Choudhury, Biplab Kumar Chakra, Tapan Kumar Chatterjee

**Affiliations:** 1 *Department of Pharmaceutical Technology, Jadavpur University,*; 3 *Department of Pharmaceutical Science and Technology, JIS University, Kolkata, India,*; 2 *Department of Pharmaceutical Technology, School of Pharmacy, International Medical University, Jalan Jalil Perkasa, Bukit Jalil, Kuala Lumpur, Malaysia.*

**Keywords:** Microsphere delivery- gum katira- toxicity study- oral delivery- optimization- Mtt assay

## Abstract

Controlled release delivery system of chemotherapeutic agents at the site of colon endorses modern drug-entrapped delivery tools, which release the entrappedagents at a controlled rate for anextended period providing patient compliance and additional protection from the degradinggastric environment. Thus, the present study was aimed to develop and optimize a novel polymeric microsphere of 5-fluorouracil (5-FU) using natural gum katira to obtain an optimal therapeutic response at the colon. Due course of experimentation, in-vivo safety profile of the gum katira in an animal model was established. Modified solvent extraction/evaporation technique wasemployed to encapsulate 5-FU in the natural polymeric microsphere and was characterized using in-vitro studies to investigate particle size, morphology, encapsulation efficiency and release of the drug from developed formulation. Formulated and optimized polymeric microsphere of 5-FU using gum katira polymer own optimal physicochemical characteristics with a fine spherical particle with size ranged from 210.37±7.50 to 314.45±7.80 µm.Targeted microsphere exhibited good cytotoxicity and also has high drug entrapment efficiency, and satisfactory release pattern of the drug within a time frame of 12 h. Finally, we foresee that the optimized polymeric gum katiramicrosphere of 5-FU could be a promising micro-carrier for efficient colon drug targeting delivery tool with improved chemotherapeutic efficacy against colon cancer.

## Introduction

Among the several routes employed for drug administration, targeted deliveries either for systemic exposure or locally to a specific site of the body, an oral route is considered the most convenient. However, drugs producing local toxicities to the gastrointestinal (GI) tract, or the unstable drugs in the gastric environment are unable to deliver via this conventional oral drug delivery system (Choudhury et al., 2017). Further, conventional oral delivery of drugs could not be able to achieve a sustained treatment for a duration of 24 h, and thus an initial bolus dose followed by maintenance of a therapeutic amount of drug to the desired site need to be delivered to achieve the therapeutic role promptly and to maintain the desired drug concentration for the desired duration (Varde and Pack, 2004). Moreover, these conventional oral dosage forms provide a bolus exposure to the drug. Therefore, to maintain the spatial placement of the therapeutic agent and governing the rate of drug delivery to the target site, drug delivery scientists are focusing on controlled release drug delivery systems. Because of which, controlled release drug delivery systems have been developed to address limitations of the conventional methods of oral drug therapy. Therefore, controlled and targeted drug delivery systems include several devices, such as a hydrogel, liposomes, polymer-based pellets, rods, disks or microparticles, where the loaded drug will be incorporated within the delivery tool to release the drug at a fixed rate for a prolonged period, even days to weeks to months (Varde and Pack, 2004). Each device consists of own advantages and disadvantages towards the delivery of drugs in a controlled manner, where polymeric microsphere persuaded to be a promising drug carrier in this field. These polymeric microspheres are consisting of biodegradable polymer matrix, where the therapeutic agent uniformly disseminated within the matrix and the loaded drug released at a sustained rate over a wide area of drug absorption during passage through GI tract without being affected by contents in the track, gastric emptying, and different transient rates (Bharaniraja et al., 2011a; Ruhidas et al., 2016). The composition of the polymer matrix of microsphere plays the critical role in designing the controlled release dosage form, where researchers are more inclining towards natural polysaccharides for their beautiful inherent properties, such as, biocompatible, biodegradable, sustainable, reliable, and inexpensive compared to other synthetic or semisynthetic products (Emeje et al., 2009). Among the natural polysaccharides, xanthan gum, dextran, chitosan, guar gum, pectin, and locust bean gum extensively used in formulation science; however, these gums remain undigested within the GI system, and degraded by the anaerobes in the colon to utilize the degraded products as their energy source (Halsas et al., 1999; Chourasia and Jain, 2004; Bharaniraja et al., 2011b). The present study has explored the natural polysaccharide gum katira in our polymeric formulations as a matrix building material. It is abundance, inexpensive and available in nature as a heteropolysaccharide from Cochlospermum religiosum, consists of L-rhamnose, D-galactose, and D-galacturonic acid at a molecular ratio of 3:2:1, together with traces of ketohexose (Jain and Babbar, 2002; Ojha et al., 2008; Bharaniraja et al., 2011b).

Chemotherapeutic agent, 5-fluorouracil (5FU) is a potential therapeutic agent for its broad spectrum of activity and clinically used in several solid tumors, viz. colorectal, breast, and head and neck cancers (Haggag et al., 2018). This 5-FU leads to the apoptotic damage of the cancer cells via interfering with thymidylate synthesis (Longley et al., 2003). Several limitations of 5-FU restrict its use, which includes variability in oral bioavailability, non-selective bio-distribution, shorter half-life, and unfavorable toxicities. However, formulation scientists have extended their researches towards effective and safe delivery of this drug via the development of nanoemulsion (Shakeel et al., 2014), liposomal delivery (Pentak et al., 2012), polymeric nanoparticle (Haggag et al., 2018), solid-lipid nanoparticle (Patel et al., 2014), etc. A polymeric delivery system using natural gum katira has been pitched in this research, where the drug will be encased in the microsphere to obtain an optimal therapeutic response at the colon. Firstly, the study is planned to determine the safety of gum katira in an animal model to progress towards the formulation development of the polymeric microsphere of 5-FU and further developed microspheres were characterized with respect to particle size, morphology, encapsulation efficiency,drug-excipient interaction, in vitro release profiles, and finally cytotoxicity of the formulation was determined using cancer cell lines. Thus, it is hypothesized that delivery of 5-FU through this gum katira microsphere dosage form will specifically avoid the gastric environment and will reach to the colon, where it will attach to the villi and release the entrapped drug for prolonged period. Such modified drug delivery may bring a potential tool to control the growth of colon cancer without producing systemic toxicities (e.g., nephrotoxicity) as evidenced by the conventional dosage forms because of high plasma concentration of the drug. 

## Materials and Methods

*Materials*

The active pharmaceutical ingredient (API), i.e., 5-FU in our experiment, was purchased from Sigma Aldrich. Ethyl cellulose was obtained from Quest Chemicals, Kolkata, India, whereas, dichloromethane, Tween-80, Span 80, hydrochloric (HCl) acid (35%), dichloromethane, potassium dihydrogen phosphate were purchased from Merck, India. Evonik Rohm, Pharma Polymers, Germany gifted Eudragit^®^RS100, and Eudragit^®^ RL100 polymer granules to conduct our research. Tri-sodium orthophosphate was obtained from LobaChemical Pvt. Ltd. India. Crude gum katira was received from Seoni District, Madhya Pradesh, India. All other reagents were of analytical grade.

*Subacute toxicity study of crude gum katira*

*Animals for the study*

The animal study was conducted in agreement with the ethical rules on animal experimentation approved by the Ethical Committee, Department of Pharmaceutical Technology, Jadavpur University (Approval No: 147/1999/CPCSEA). Committee for the Purpose of Control and Supervision of Experiments on Animal (CPCSEA) guideline was followed for the animal handling and care throughout the probationary period. All the animals were purchased from a registered breeder which were then acclimatized at 25±2°C, and 50±5 % relative humidity with 12 h light/12 h dark cycle and animals were retained in polypropylene cages provided by standard pellet diet (Hindustan Lever Ltd., Mumbai, India) and water ad libitum (Jaisankar et al., 2016).

*Toxicity study*

The OECD guideline was followed to conduct a14 days subacute toxicity study using male Wistar rats weighed around 120-130 g (Oecd, 2001). Animals were divided into four groups with six animals in each. Since, during the experiment for acute toxicity study, gum katira was found to be safe in experimental animals up to a dosage level of 2 gm/kg body weight, the selection of the dosage in this toxicity study was made based on the maximum content of gum katira during treatment of formulated microparticles. Therefore, the gum katira solution at 150, 300 and 400 mg/kg body weight dose were orally administered every day to the particular group of animals for a period of14 days, whereas the animals in control group received vehicle (vehicle control). The toxic manifestation concerning the appearance, body weight, behavioral changes, mortality, stool were observed regularly. After the duration of 14 days, all the surviving animals were kept in fasting condition overnight, and few animals were anesthetized to collect the heparinized blood samples for hematologic parameters determination and non-heparinized blood samples for biochemical parameters. Resting animals were sacrificed by euthanasia where the internal organs, such as kidney and liver, were collected and preserved in 10% formaldehyde solution for histopathological examinations. 

*Microbial load study*

The bacterial and fungal load of the samples were led by the standard pour plate method. Sterilized Petri dishes with soybean-casein digest agar medium (suitable for bacterial growth), and potato-dextrose-agar medium (suitable for fungal growth) were poured aseptically. The sample of fresh gum katira and ten months older sample of the same component were incorporated aseptically onto the respective Petri dishes. After solidification of the media, the Petri dishes were placed in the incubator maintaining 37°C temperature for 24 h. The total number of colonies were counted by the Coulter Counter method. The fungal count was also conducted by using the above-said method (Farag Zaied et al., 2007).

*Moister content of gum katira*

Carl Fischer titration method was used to investigate moisture content of gum katira, where the gum powder was dispersed in methanol and further stirred for 5 min to remove water. Finally, it is titrated with standardized Karl Fischer reagent to reach the endpoint. The formula was used to calculate the moisture content of the polymer:

$\mathrm{moisturecontent}\left(\%\right)=\frac{V_{1}\times W_{e}}{S_{w}}\times 100$

Where V1: volume of Karl Fischer reagent used, We: water equivalent, and Sw: the weight of the sample. (Water equivalent(We) is 5.2)

*Formulation development*

*Preparation of 5-FU stock solution*

The stock solution of 5-FU (100 µg/mL) was prepared by dissolving 10 mg of 5-FU in 50 mL of distilled water; further, volume was made up to 100 mL in a volumetric flask.

*Preparation of 5-FU loaded gum katira microsphere*

The double emulsion solvent evaporation technique (W1/O/W2emulsion) is employed in our experiment to encapsulate hydrophilic drug, where, the limitations of a single O/W emulsion, to entrap only lipophilic drugs can be avoided.

Therefore, 5-FU loaded gum katira microspheres were prepared by W1/O/W2 emulsion solvent evaporation technique. Gum katira (50 mg) was mixed with 4 mL of aqueous solvent with continuous stirring for half an hour using magnetic stirrer at 35°C to form a homogeneous solution. A measured quantity of 5-FU was added to the solution of gum katira and continue stirring for another half an hour. Subsequently, a solution of Eudragit^®^RS100 and Eudragit®RL100 (7:1; w/v)(700mg of Eudragit^®^RS100 and 100mg of Eudragit^®^RL100 were added in 30 mL of dichloromethane (DCM)), acryflow and Span 80 was prepared and instantly transferred to the homogenizer tube. The homogenizer was subjected to a rotational speed at 4500 rpm, while the drug solution was poured drop by drop into it through a 20 gauge needle. The homogenization process was continuedfor5-10 min to prepare the primary W1/O emulsion. Later, the prepared emulsion was added dropwise to 100 mL of acidic aqueous solution (pH-4.0) using a 16 gauge syringe to form the W1/O/W2 emulsion with continuous stirring at 700 rpm for 2.5 h. The resultant microspheres formed were washed with distilled water followed by filtration, air-drying for 24 h, and finally stored in desiccators for further uses.

*Morphological analysis using scanning electron microscopy (SEM)*

The texture of microspheres was determined by using SEM (CARL ZEISS EVO 18 special edition machine) and also examined the morphology of the cross-sectioned surface of the prepared microsphere. SEM analysis was performed with the platinum coating, which was performed using QUORUM Q150 TES. 

*X-ray diffraction (XRD) study*

XRD analysis provides about the information of crystalline or amorphous nature of the incorporated drug in the developed microspheres. XRD study was performed by X-ray diffractometer of model Ultima-111, Rigaku (Japan), using copper target slide (10 mm), where5 mg sample was placed on the holder. The diffractogram of the samples was recorded in the range of 2*θ* = 1–100° at a scanning rate of 5°/min.

*Fourier transforms infrared (FTIR) spectroscopy*

FTIR analysis executed to find out for any chemical interactions between the drug molecule, and other ingredients used in the microsphere preparation, where 2 gm of the sample was crushed and then mixed thoroughly with 10 gm of fine alkali halide (KBr) powder and pressed into a transparent pellet using a hydraulic press. The formed pellets were then individually scanned using FTIR spectrophotometer (IR-Prestige-21, Shimadzu, Japan) at a spectral region of 4,000–400 cm^−1^ with a resolution of 4 cm^−1^ and 16 scans were obtained for each spectrum.

*Percentage Yield*

Theoretical yield is the total weight of the raw materials used for preparation, whereas practical yield represents the weight of the microsphere, which was practically obtained (Belgamwar et al., 2011).

$\mathrm{percentage of yield}=\frac{\mathrm{practicalyield}}{\mathrm{theorecticalyield}}\times 100$

*Effect of process variables*

For the preparation of the microspheres, various factors or variables influence the output of the product. Some of those variables were important to be observed, which include the concentration of gum katira, stirring speed during formulating of secondary emulsion, the ratio of incorporating co-polymers, processing temperature, inner phase volume, and concentration of span 80. The range of all these variables observed within the boundaries of our laboratory condition are listed below:

*Estimation of drug entrapment efficiency (EE)*

The percentage EE (%EE)within the microspheres was determined spectrophotometrically. Drug-loaded core-shell microspheres (10 mg)were crushed, powdered and dissolved in 5 mL of DCM and the solution was stirred for 10 min using a magnetic stirrer for the complete dissolution of the polymer in DCM. Further, 10 ml of methanol was added to the resultant solution, followed by magnetic stirring for 2 min at 40-45ºC and filtered. The absorbance of the final solution was measured at 270 nm using double beam UV-Visible spectrophotometer (UV1, ThermoSpectronic, Great Britain) against methanol as blank.

The % EE was calculated using the following equations: 

$\mathrm{percentage entrapment of liciency}=\frac{w_{t}-w_{f}}{w_{t}}\times 100$

Where, Wt is the total initial amount of 5-FU incorporated during formulation development, and Wf is the amount of free drug in the solution after formulating the drug entrapped microspheres (Singhavi et al., 2017).

*Determination of particle size*

The particle size of prepared microspheres was determined by using Zetasizer instrument(DLS-nano ZS, Zetasizer, Malvern Instruments). A small quantity (10 mg) of microspheres was dispersed in Milli-Q water (Milli-Q, Merck Millipore, Billerica, MA, USA) containing Tween 80 (0.2%, w/v) by vortexing and then sonicated and placed in a cuvette for measurement of particle size.

*In vitro dissolution study*

In vitro release of 5-FU from the 5-FU loaded microspheres was carried out using a digital USP type II dissolution test apparatus (Lab India DS 8,000 USP). Dried microspheres (50 mg) were suspended in 500 mL of either 0.1NHCl solution of pH 1.2 or phosphate buffer saline solution of pH 7.4. The dissolution media was maintained at 37±0.5 under continuous stirring at 50 rpm. At predetermined intervals, the sample was withdrawn, and the concentration of released 5-FU in the media was determined by analyzing at 270 nm using a double beam spectrophotometer (UV1, ThermoSpectronic, Great Britain). The cumulative release of 5-FU was determined using the standard calibration curves of the drug in respective dissolution media (0.1 N HCl - pH 1.2; phosphate buffer saline - pH 7.4), respectively (Maiti et al., 2009; Ahmadi et al., 2018).

*Measurement of swelling index*

Rate of swelling/hydration of 5-FU loaded gum katira microspheres were evaluated to study the extent of water uptake and drug release mechanism from the hydrated microsphere. Accurately weighed microspheres were placed on Petri dishes containing dissolution media (for the first 2 h in 0.1N HCl solution (pH 1.2), followed by 10 h in phosphate buffer (pH 7.4)). Swollen microspheres were withdrawn at the specified time from the medium and weighed in an electronic balance after removing the excess surface water using bloating paper. The same process was continued for 12 h to calculate the percentage of swelling using the formula mentioned below (Kaur et al., 2017):

$\mathrm{\% Swelling}=\frac{S_{t}-S_{o}}{S_{o}}\times 100$

Where S_0 _is the initial weight of microspheres, and St is the weight of hydrated microspheres at the particular time. 

*Cell cytotoxicity assay of 5-FU loaded microsphere*

*Cell line and cell culture*

HCT-116 (human colon cancer cell line), MCF-7 (breast cancer cell line) and HeLa (cervical cancer cell line) cells were obtained from Chittaranjan National Cancer Research Institute(, Kolkata, India. All the cells were incubated separately with 5% fetal bovine serum (FBS) containing penicillin and streptomycin at a concentration of 100 μg/mL each 37°C in a humid atmosphere (5% CO_2_; 95% air). Cells were harvested by short incubation in 0.02% (w/v) EDTA in PBS. The cells were maintained routinely in subcultures in tissue culture flasks.

*Cell cytotoxicity assay*

The cytotoxicity of free 5-FU, 5-FU loaded microsphere, and blank microspheres were ascertained by MTT assay which is measuring the activity of the mitochondrial succinic dehydrogenase enzyme that converts MTT, a tetrazolium salt, into formazan crystal (Lee and Low, 1995; Waheed et al., 2013). Investigations were performed in 96-well flat bottomed culture plates (BD Biosciences, USA). MTT was dissolved in phosphate buffered saline (PBS) at a concentration of 5 mg/mL. Next, to this, different concentrations of the sample (0.5, 5 and 10µg/ml) were added, and the plate was incubated for a period of 24 h. Following this, 20 μL of MTT solution, prepared as above, was added to each well and incubated for four h at 37°C, the culture medium was removed, and the formazan crystals were dissolved in 200 μL DMSO. The absorbance of formazan dye was measured at 570 nm using a microplate reader (Tarsons, India, Cat. No: 980040). The negative control was the cells without treatment (MTT group) as well as cell incubated with blank microsphere. No positive control was used in this experiment; the cytotoxicity of 5-FU loaded microspheres was compared with free 5-FU, a standard antitumor drug. The amount of formazan produced resembles with the number of live cells (Hemaiswarya and Doble, 2013). Cell viability was calculated using the following equation:

$\mathrm{cellviability\%}=\frac{\mathrm{Absa}}{\mathrm{Absc}}\times 100$

Where Absa is the absorbance of the sample treated with 5-FU (free or microencapsulated), and Absc is the absorbance of the control group cell.

## Results

*Toxicity study*

Investigation of toxicity study is conducted to examine the safety profile of the material through different non-clinical toxicity studies before being administered in human subjects. Here, the toxicity study of gum katira was conducted under the OECD guideline and the guidelines provided by the animal ethics committee. Hematological findings of the gum katira treated animals with doses of 150, 300, and 400 mg/kg body weight depicted a maintained hemoglobin count towards the normal levels. Concurrently, the RBC count, WBC count, platelet count, and differential counts have also been found within the normal range in animals in a gum-treated group when compared with the control group (Table 1). Simultaneous estimation of biochemical parameters in the separated serum of the experimental animals demonstrated that SGOT, SGPT, ALP, and bilirubin were not likely to change as compared to the control group. Thus, from the above verdicts, it can be inferred that treatment with gum katira at different doses did not change the hematological and biochemical parameters significantly. 

**Figure 1:**
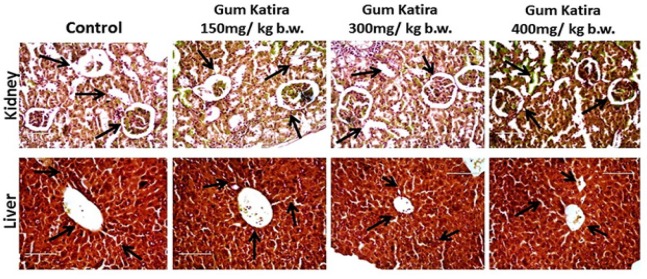
Histopathological Sections of Liver and Kidney Slices of Swish Albino Rats Stained with Hematoxylin and Eosin and were Examined under the Light Microscope at 100x. Liver sections of treated and untreated groups were exhibiting the presence of all the standard features, including circular hepatic portal vein and branch of the hepatic artery, as marked by arrows. Simultaneously, kidney sections demonstrated normal glomerulus, normal basement membrane, capillaries collecting ducts, tubules, ascending, and descending loops without any inflammatory cells as compared to the normal healthy rat (marked by arrows)

Histopathological examination of the liver and kidney of the experimental animal were also assessed. Hematoxylin and eosin stained sections of liver slices of healthy rats displaying the incidence of all the standard and regular structure, which includes a branch of the hepatic artery, and circular hepatic portal vein, as shown by arrows. The hepatocytes showed prominent nuclei (marked by arrow) and the tissue section included hepatic sinusoids. On the other hand, all of the regular features as mentioned above were available in the treated animals, and thus the negligible amount of phenotypic alteration and altered hepatocyte population were found in animals treated with gum katira at the different dose (Figure 1). Consequently, on animals with an escalated dose of gum katira, 150, 300 and 400 mg/kg body weight, the overall and cellular features have resembled healthy animals. The histogram demonstrates the presence of regular central vein, normal hepatocytes, branch of the bile duct and hepatic artery, demonstrating the superiority of the same (Figure 1). 

On the other side, histopathological examination of the kidney sections of the animals in control group displayed regular and standard assembly of glomerulus bounded by the Bowman’s capsule, proximal convoluted tubules and distal convoluted tubules devoid of any inflammatory changes (Figure 1). The groups that were treated with gum katira at doses of 150, 300 and 400 mg/kg body weight demonstrated normal glomerulus, normal basement membrane, capillaries collecting ducts, tubules, ascending and descending loops without any inflammatory cells as compared to the vehicle control group (Figure 1, marked by arrow). From the above findings of blood hematological, biochemical, and histopathological parameters, it can be depicted that gum katira is safe to be administered for in vivo use.

**Table 1:** Effect of Gum Katira at Different Doses on Blood Hematological and Biochemical Parameter

Parameters	Control	Treatment with gum katira (/kg body weight)		
		150 mg	300 mg	400 mg
Haemoglobin (gm%)	14.31±0.33	14.30± 0.44	14.35±0.16	14.35±0.15
RBC (10^6^/µl)	8.74±0.037	8.72±0.059	8.73±0.223	8.75±0.036
WBC (10^3^/μl)	13.63±0.054	13.52±0.041	13.55±0.043	14.01±0.021
Neutrophil (10^3^/μl)	20.73±0.658	20.01±0.348	20.92±0.521	20.96±0.134
Monocyte (10^3^/μl)	2.31±0.343	2.32±0.063	2.38±0.224	2.71±0.151
Lymphocyte (10^3^/μl)	71.66±0.343	71.69±0.547	71.79±0.642	71.89±0.351
Eosinophil (10^3^/μl)	2.33±0.135	2.34±0.786	2.54±0.132	2.61±0.634
Platelets (10^3^/μl)	1,241.05±1.038	1,248.10±1.821	1,251.02±0.713	12,59.06±1.076
SGOT (U/L)	90.99±0.335	92.11±0.432	93.42±0.542	94.00±0.341
SGPT (U/L)	34.63±0.542	34.32±0.642	34.19±0.444	34.14±0.465

Each point represents the mean ± SD. (n= 3 animals per group).

** p < 0.01 statistically significant when compared with the normal saline group

**Table 2:** Effect of Process Variables on the Properties of 5-FU Loaded Gum Katira Microspheres

Processing variables	Prepared batches	Entrapment efficiency (%)	Average particle size(µm)	% drug release at 12 h
Gum katira(mg)				
50	FA1	59.45±3.18	210.37±7.50	80.58±2.46
** 75**	**FA2**	**77.25± 4.25**	**247.54±8.30**	76.38±2.35
100	FA3	68.13±5.25	283.85±10.12	68.73±1.96
125	FA4	63.37±4.13	314.45±7.80	65.25±2.19
Span 80 (% v/v)				
0	FB1	33.15±2.35	310.67±9.85	86.73±2.27
** 0.5**	**FB2**	**73.25±6.15**	**260.35±6.50**	78.55±1.53
1.5	FB3	28.37±5.25	-	83.27±1.37
Stirring speed (rpm)				
600	FC1	58.63±4.45	290.85±6.45	69.85±3.35
** 800**	**FC2**	**79.71±6.01**	**255.25±1.35**	79.35±1.97
1,000	FC3	63.15±4.25	230.75±6.37	75.54±1.53
Internal aqueous phase volume (Ml)				
2	FD1	53.19±3.35	237.25±3.37	74.53±2.25
** 4**	**FD2**	**76.53±4.15**	**249.77±8.27**	80.15±1.27
8	FD3	63.85±6.27	280.93±6.25	79.73±2.15
Co-polymer ratio: Eudragit® RS100: Eudragit® RL100				
1:00	FE1	73.58±2.31	293.75±6.23	64.93±3.37
** 7:01**	**FE2**	**76.75±3.33**	**261.27±8.31**	83.57±2.93
1:07	FE3	63.23±5.41	247.93±6.63	80.63±3.15
0:01	FE4	55.63±3.19	221.17±3.37	69.53±1.97
Processing temperature				
25	FF1	68.73±5.23	240.75±8.35	76.53±1.35
** 35**	**FF2**	**73.19±4.75**	**246.35±7.75**	79.92±1.17
45	FF3	77.27±3.75	251.90±6.65	73.25±3.11

Values are expressed as mean ± standard deviation (n= 6); Bold values are the selected processing variables for final formulation.

**Table 3:** Physical Characteristic of Optimized 5-FU Loaded Microsphere

Percentage yield(%)	Drug entrapment efficiency(%)	Cumulative drug released up to 12 h (%)	Diameter of the microsphere(µm)
91.73±2.15	76.53±1.79	82.63±3.15	258.37±5.22

Values are expressed as mean ± standard deviation (n= 6).

**Figure 2:**
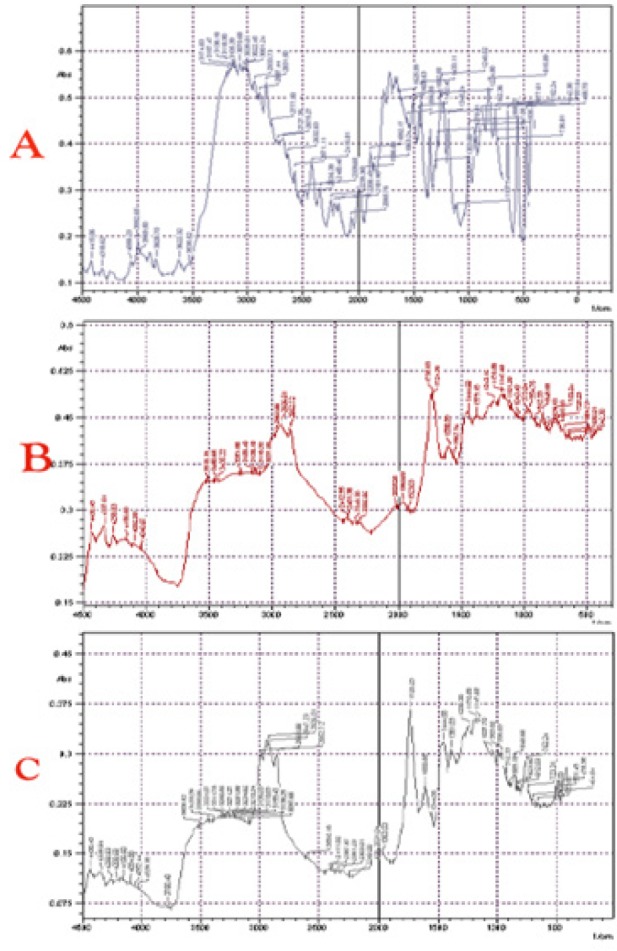
FTIR FTIR Spectrums of 5-FU (A), Blank Microsphere (B), 5-FU Loaded Gum Katira Microsphere (C)

**Figure 3:**
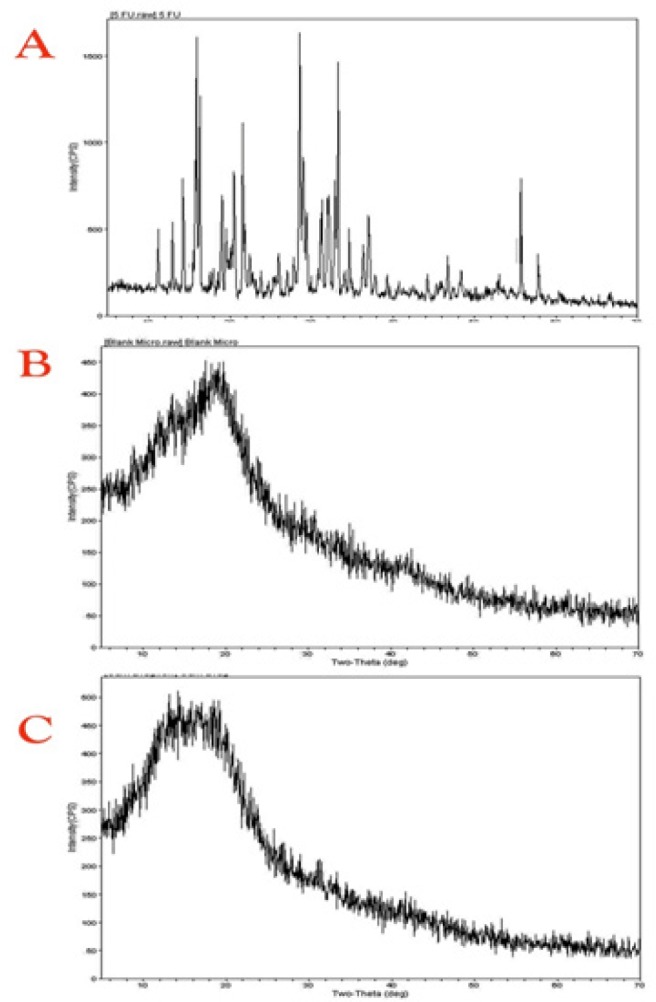
XRD Patterns of 5-FU (E), Blank Microsphere (F), and 5-FU Loaded Gum Katira Microsphere (G)

**Figure 4:**
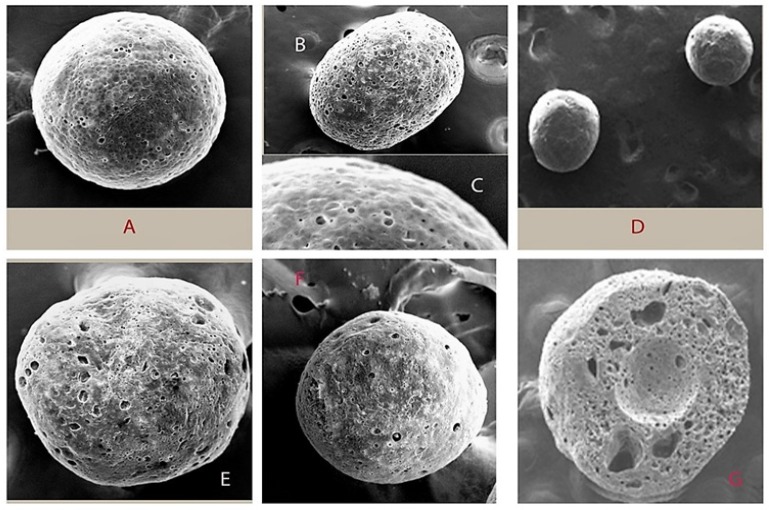
Morphology of Microsphere during Processing Variable: (A) change of gum katira concentration (surface became smoother and increase in diameter with increased quantity of gum); (B) changing quantity of span 80 (elongated microsphere without span 80);(C) surface of the microsphere;(D) effect of change of stirring speed (low, stirring speed produce smooth and small microsphere);(E) effect of copolymer ratio (larger microsphere produced with increased in concentration of RS 100);(F) change in temperature (at low temperature produced microspheres were highly porous with rough surface); (G) Cross section of the microsphere. All pictures were taken by using QUORUM Q150 TES at 300X magnification

**Figure 5:**
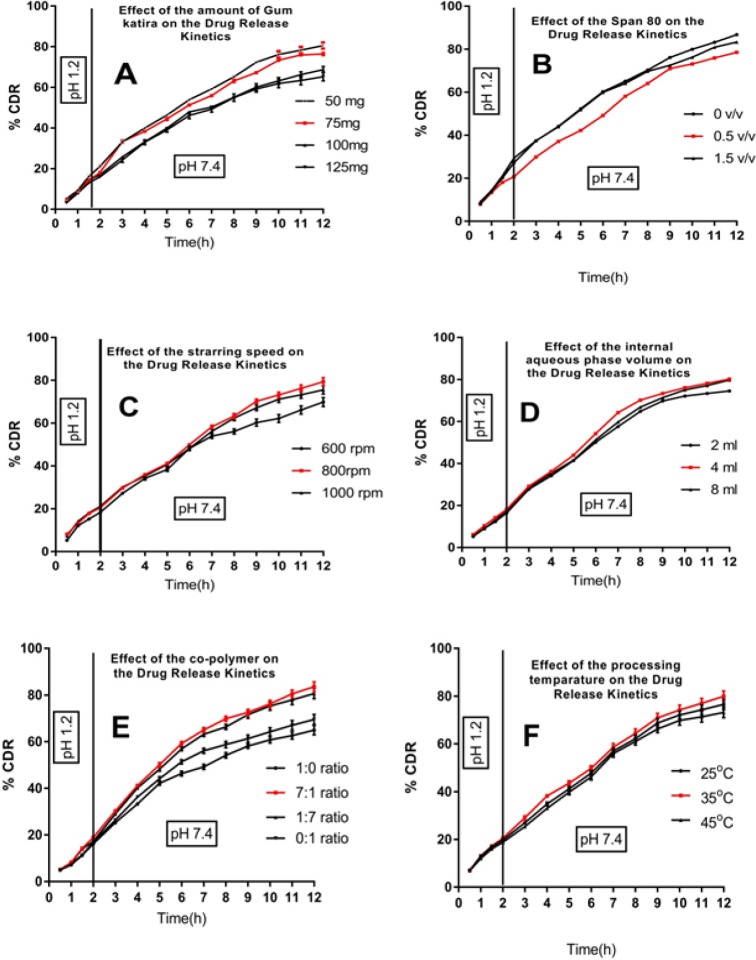
Effect of Processing Variables on the Cumulative Drug Release Profile of 5-FU Loaded Gum Katira Microsphere. Release profile of drug on (A)variation in of gum katira content;(B)variation of span 80 percentage;(C) variation of stirring speed;(D)variation of internal aqueous phase volume;(E)variation of copolymer ratio; and on (F)variation of processing temperature

**Figure 6:**
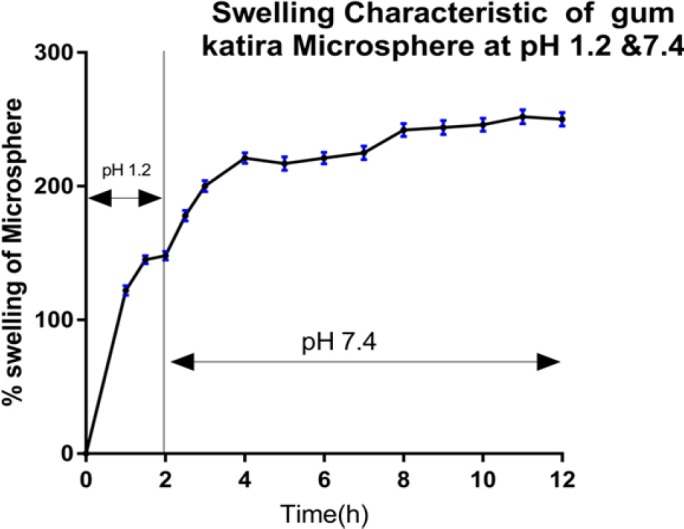
Swelling Characteristic of 1% Gum Katira Microsphere at pH 1.2 Solution (First 2h) Followed by pH 7.4 (last 10h)

**Figure 7:**
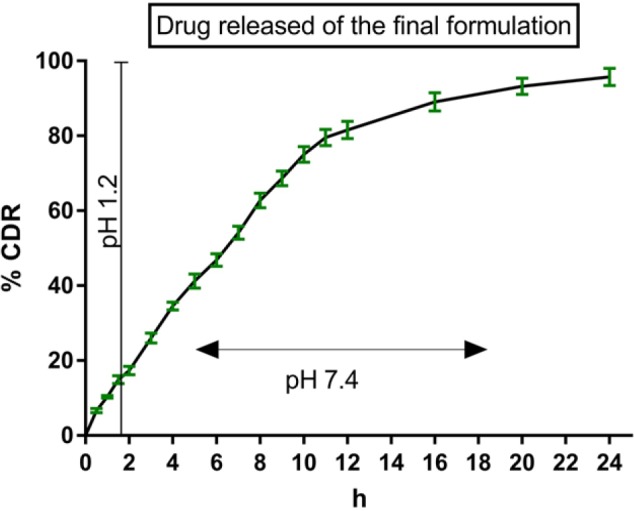
*In vitro* Release Profile of the Optimized Formulation. Where processing parameters were gum katira(75mg), Stirring Speed (800rpm), Copolymer (7:1), Processing Temp (35°C), Aqueous Phase Volume(4ml), Concentration of span 80 (0.5%).

**Figure 8:**
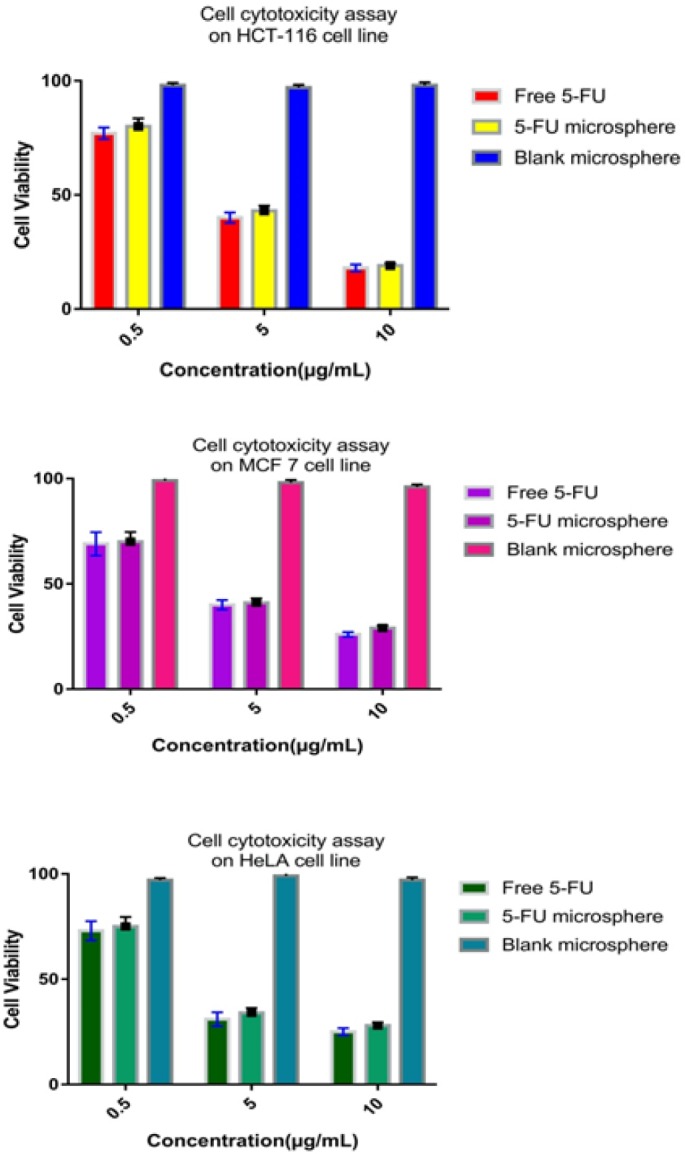
Cell Viability Percentage of Free 5-FU, 5-FU Loaded Microsphere, and Blank Microsphere on HCT-116, MCF-7and HeLa Cell Line, in 24 h. 5-FU-Loaded Microspheres Compared with Free 5-FU in the Same Concentration

*Moisture content determination*

Moisture content determination of gum katira powder using Karl Fisher reagent revealed 12.76 ±0.91% water content in the powder. It is well known that the gummy recipients with high moisture content could influence the physical and chemical stability of the incorporated ingredients, including the active drug substance. Further, it can also degrade the formulation as well. According to the pharmacopeia and WHO guidelines, the moisture content of natural polysaccharide used as an excipient should be ≤15% (Meka et al., 2012). Therefore, the use of gum katira could be satisfactorily employed in the development of our novel pharmaceutical formulations without disturbing the stability concern of the product.

*Microbial load study of obtained gum katira*

The outcome of microbial load study of the fresh and old samples was led by the pour plate method. Following incubation, for a period of 24 h at 37°C, the observed microbial count of the two test samples was 280 CFU/g and 310 CFU/g for the fresh and old samples respectively. Concurrently, the fungal count of the samples was recorded as 69 CFU/g and 77 CFU/g for the fresh and older samples of gum, respectively. The findings of our results are following the limits provided in Indian Pharmacopoeia (4th edition, 1996), where the microbial and fungal loads for a sample is limited to 1,000 CFU/g and 100 CFU/g, respectively (Joshi et al., 2017).

*Interaction study between polymer and drug*

*Fourier transforms infrared (FTIR) spectroscopy*

An interaction between the API and natural polymer was analyzed by comparing the FTIR spectrum of optimized formulations with the spectrum of pure drug and polymer. Representation of FTIR spectrum of 5-FU, 5-FU loaded gum katira microsphere, and blank microsphere was made in Figure 2 (A, B, and C), which showed –NH bond stretching between 3,000 and 3,500 cm^-1^ in the spectrum of 5-FU. In the microsphere where the 5-FUwas entrapped with gum katira, this bond was found to occur at 3,500 cm^-1^. The –OH of gum katira made an overlapping with the –NH band of the 5-FU in the spectrum. Further, C=O stretching bands were also found in the drug-loaded microspheres, blank microsphere, and the pure 5-FU at 1,735 cm^-1^, 1,737 cm^-1^, and 1,625 cm^-1^ respectively. 5-FU loaded gum katira microsphere, blank microsphere and pure 5-FU provided peaks due to the presence of C-H groups at 2,926 cm^-1^, 2,927 cm^-1^, and 2,933 cm^-1^, respectively. The C-F stretching bands of the 5-FU molecules provided peaks at almost similar wavelengths, at 1,242 for the drug-loaded microsphere and 1,246 for the pure drug, suggesting an interaction-free formulation between the API and polymer.

*X-ray diffraction (XRD) analysis*

Alternatively, XRD study reports of API, 5-FU loaded gum katira microsphere, and blank microsphere was presented in Figure 3 (A, B, and C). A sharp peak obtained in the XRD pattern of the pure drug represents the crystalline nature of the pure drug. Powder crystalline theory revealed that crystalline substances could provide clear peak structure because of the arrangement of molecules in their inner framework with the atoms and ions provide a stable network in a three-dimensional configuration, which can provide a regular diffractogram from the crystalline structure resulting in sharp peaks. However, the gum katira microsphere produced the peak of diminished intensity, which shows shifting of crystallinity characteristics of 5-FU towards amorphous nature, because the amorphous structure of the molecule failed to provide a sharp peak of the drug molecule (Abdelrazek et al., 2018). 

*Process variables*

Most widely adopted practices for microsphere preparation is the solvent extraction or evaporation techniques, which formulate the desired formulation with altered drug release profile. However, the classical solvent extraction or evaporation techniques are limited to incorporate lipid-soluble compounds through the process of emulsification of drug and polymer solubilized organic solution in a continuous aqueous phase. Thus, modified methods are in use to incorporate miscible water drugs, through incorporating in the aqueous phase as a saturated solution (Bodmeier and Mcginity, 1987) or formulating multiple emulsion (W1/O/W2) (Ogawa et al., 1988). Alternatively, an oil-in-water-in-oil solvent evaporation/extraction process is also available in the pharmaceutical research where another oil phase substitutes the continuous aqueous phase. The water-immiscible drug can be incorporated in second oil, to introduce the water-immiscible drugs into the microsphere (Bodmeier and Paeratakul, 1994).

Involved processing parameters plays an essential role in this modern W1/O/W2 solvent evaporation technique for microspheres preparation. Thus, the focus of our present work was directed towards systemic investigation of the parameters, which have a direct influence on the properties of microspheres.

*Effect of varying concentration of gum katira*

The formulation development study was aimed to prepare a polymeric microsphere with a high drug EE with intended drug release kinetics. During formulation development, 5-FU loaded microspheres were formulated using a different concentration of gum katira (50, 75, 100 and 125 mg) to investigate surface morphology, the %EE, release behavior of the entrapped drug at pH 1.2 and pH 7.4. The %EE of microsphere was found to be varied significantly due to the different concentration of the gum katira, which ranged from 59.45±3.18 %to 79.25±4.25%. Increasing the concentration of gum in the composition increases the diameter of the formulated microsphere. The volume of the organic phase used in the developmental process was kept constant. Therefore, it could be postulated that increase in the concentration of gum in the composition may lead to increase the frequency of collisions between particles, resulting in the amalgamation of semi-formed particles, and finally increase the diameter of the final microsphere (Bile et al., 2015). Alternatively, increased concentration of gum katira as a drug release modifier in a fixed volume (4mL) of the inner phase affected the particle size of the microsphere (Table 2). The high viscosity of the W1 phase minimized the possibility of the spontaneous breakdown of W1 phase into smaller droplets, thereby formed larger microspheres (Bee et al., 2018). However, when observed under scanning electron microscopy, the surface of the microsphere perceived smoother and spherical with the increase in gum concentration (diameter 314.45±7.80 with 125 mg gum katira) (Figure 4A).

However, with the increase in gum katira within the inner phase reflected by an increase in %EE up to a certain limit (50mg) beyond which it was decreased. The higher viscosity of W1 phase results in the formation of the homogeneous emulsion with numerous internal droplets in the W1/O emulsion that aggravates the leakage of inner core materials to the external aqueous phase in comparison to the low viscosity of W1 phase that inhibits the leaching of the drug from inner phase to external phase (Bee et al., 2018). 

The drug release profile of microsphere formulations with a variable amount of gum katira in W1 phase was significantly different. Increase in the amount of gum katira in aqueous phase retarded the release of incorporated drug in the dissolution media (Figure 5A). The highly viscous solution inside the inner phase of the microsphere may provide resistance to drug diffusion from the inner core to external dissolution media (Sinha et al., 2004).

*Effect of varying span 80 concentration in the oil phase*

Span 80 is a nonionic dispersing agent with a hydrophilic-lipophilic balance value of 4.3 and is mainly used to stabilize the w/o primary emulsion. The role of Span 80 is expected to have a high disparity for the present emulsion system by reducing the surface tension at the interface(Nesterenko et al., 2014). Three formulations were formulated where one is devoid of Span 80, and the other two were formulated using different concentrations of Span 80 (0.5% and 1.5%,w/v). The microspheres formulated without surfactant in the oil phase became elongated; however, at the intermediate concentration, the microspheres were spherical and individual (Figure 4B). Further increase in the concentration of Span 80 lost their individuality and existed as aggregates (data not shown). While the microspheres showed monomodal size distribution at 0.5% (w/v) of Span 80. The mean diameter of the microspheres was 260.35±6.50 μm with the use of Span 80 (0.5%,w/v). 

The %EE of microspheres was higher at the intermediate concentration of emulsifier than those observed at other concentrations (Table 2). Since the %EE and particle size distribution of the formulated microparticles are significantly connected to the stability of the primary emulsion, this can be illuminated by the Span 80’s tension-active characteristics, which stabilized the rest emulsion and prohibited the quick coalescence of the dispersed droplets (Jiao et al., 2002).

Further, Figure 5B represents the influence of Span 80 on the release characteristics of drug-loaded microspheres in both alkaline and acidic dissolution media. The microspheres prepared with 1.5% Span 80 or without Span 80 showed faster drug release in both media. Comparatively, a slower drug release profile was achieved at the intermediate(0.5%) concentration of Span 80. Decreasing of surface tension would not be reflected in the absence of surfactant, which further hindered the division of aqueous inner phase into finer droplets, caused slower precipitation of the polymer, created possibilities for the drug to be present over the surface of semi-formed microspheres, and thus led to quicker drug release. On other hands, at higher concentration of Span 80, the particles became smaller, thereby increasing the active surface area for faster drug dissolution (Jain et al., 2000; Mundargi et al., 2008).

*Effect on stirring speed on microspheres preparation*

The impact of agitation during final emulsification throughout the production of drug-loaded microspheres was also studied. Different stirring speed maintained during the preparation of microsphere formulations were 600, 800 and 1,000 rpm. Changing stirring speed from 600 to 1,000 rpm caused a marked variation in average particle size as well as %EE (Table 2) and morphology of the microspheres. The progressed stress generated in the emulsion with an increase in the speed of mechanical stirrer leads to dividing the droplets of the emulsion and finally, small particles formation (Barkat Ali Khan et al., 2011). The formation of smaller emulsion droplets facilitated drug diffusion out of the microspheres before they harden and resulted %EE became lower (Zhou et al., 2005), when the agitation speed was near about 1,000 rpm, a minor change in morphology was inspected, mainly some channel was formed. Such generated channels affected the drug release characteristic. The small particles manifested faster drug release behavior in comparison to large particles. Vast surface area and porous surface structure of microsphere particles are reasonable causes for such behavior (Lamprecht et al., 2003). Low stirrer speed formed a smooth and spherical microsphere (Figure 4D). The stirring speed of 800 rpm formed the microspheres with good drug release behavior, shape, and size (Table 2).

*Effect of aqueous phase volume in the preparation of microspheres*

Here, 5-FU loaded gum katira microsphere formulations were formulated by a double-emulsion solvent evaporation method using different amounts of inner phase volume to optimize the %EE, particle size, and drug release profile. Increase in the volume of the aqueous phase caused an insignificant increase in the mean diameter of the microspheres. It was observed that %EE was higher in 4 mL of the aqueous phase in comparison to 8 mL of the aqueous phase in the double emulsion system (Table 2). It can be explained that the lower volume of the inner phase (W1) reflects a higher concentration of gum, which ultimately produces a viscous inner layer. Therefore, it may form a relatively thick layer on the organic phase (O), which can act as a barrier towards the diffusion of the entrapped drug to the external acidic aqueous phase and thereby decrease in drug release (pH-4.0) (Matos et al., 2014).

Morphological analysis has shown that the variation in the internal aqueous phase volume did not influence the spherical shape of the microspheres (micrographs not shown). An increase in inner phase volume tended to produce larger microspheres which were due to the increase in the number of dispersing droplets in a fixed volume of the organic phase (DCM), which may result in coalescence among the dispersed droplets to become a microparticle with bigger diameters (Lai and Tsiang, 2005). It has also been observed that the microspheres formulated with lower internal phase volume (4 mL) released the entrapped drug at a slower rate in the dissolution media than the microspheres formulated with higher internal phase volume (8 mL) (Figure 4D). Crotts and Park reported in his research that the porosity of the developed microsphere increased with an increase in the volume of the inner aqueous phase in the primary emulsion, which effectively increases the release pattern of the entrapped drug (Crotts and Park, 1998). Therefore, our findings were in line with the previous finding in the same field.

*Effect of copolymer ratio in the preparation of microspheres*

Eudragit, such as RS100 and RL100, exhibit a dissolution threshold slightly above pH 7.2. These properties of the above polymer resins are uniquely centered on its arrangements of the chemicals, methyl methacrylate, methyl acrylate, and methacrylic acid (Lamprecht et al., 2003). Due course of our formulation development, microsphere formulations were prepared using a different ratio of Eudragit^®^RS100 and Eudragit^®^RL100 (1:0, 7:1, 1:7, 0:1) and investigated the drug entrapment efficiency, particle size, and the drug release pattern. The selected combination of Eudragit^®^RS100 and Eudragit^®^RL100 in the microsphere preparation provide a suitable matrix structure aimed for better drug release profile. The drug entrapment was non-significantly increased (73.58±2.31 to 76.75±3.33) with increasing concentration of Eudragit^®^RS100 (Table 2). The contingent reason may be the higher amount of Eudragit^®^RS100 in DCM enhances the viscosity of organic phase, and it has a tendency to resist the migration of drug from inner phase to external aqueous phase (Rafati et al., 1997).

Morphological studies showed the denser microspheres with an increased quantity of Eudragit^®^RS100 (Figure 4E). It can be said that a high amount of Eudragit^®^RS100 in organic solvent led to an increase in the frequency of collision and resulted in the fusion of semi-formed particles, and formed larger microsphere (Sato et al., 1988).

The different ratio of co-polymer influenced the drug release kinetics significantly. It was noticed that drug release rates from Eudragit^®^RS100 coated microspheres were very slow and incomplete, whereas Eudragit^®^RL100 coated microspheres showed a relatively higher drug release rate (Figure 5E). The relative increase in release rate could have happened because of the presence of more number of quaternary ammonium groups in Eudragit^®^RL100 (Kawashima et al., 1989).

*Effect of temperature in the preparation of microspheres*

The drug entrapment efficiency of 5-FU loaded microspheres at 35ºC was higher when compared to microspheres formulated at 25ºC. It can be assumed that higher the rate of evaporation of the organic solvent at high temperature led to fast solidification of polymer and resulted in the high %EE (Li et al., 1995). The microspheres were hardened rapidly, and the surface became rough at a high temperature (35ºC)due to the rapid phase separation and evaporation of DCM during the shrinking and hardening stage (Yang et al., 2000) (Figure 4C). The rough surface of the microsphere is beneficial for enhancement of mucoadhesive property of the dosage form with the villi in colon wall that provides a suitable environment for the extended drug release (Duan et al., 2016).

Further increase in temperature, up to 45 (above DCM boiling point, 39.6), detected by very rapid evaporation of DCM and thus the porosity of the formulated microsphere lost their normal behavior.

It was also observed that the particle size of the microspheres was increased with the increasing of the processing temperature. The drug release characteristic of microsphere formulation prepared at different processing temperature had been displayed in Figure 4F. 

*The physical characteristic of optimized 5-FU loaded microsphere*

The final formulation with the optimized parameters based on outcomes through process variable patterns, the diameter of the microsphere was found to be 258.37±5.22 µm. Finally, within the limit of our experimentation, the percentage yield for the optimum formulation of gum katira microsphere was found to be 91.73±2.15% with drug entrapment efficiency 76.53±1.79%, and cumulative drug release at 12 h was 82.63±3.15% (Table 3).

*Swelling index*

Despite the nature of the entrapped drug in a polymeric structure, the hydrophilic polymers swell directly proportional to the quantity of polymer in the formulation. Polysaccharide matrix shows initial rapid swelling (due to stress induced by ingresses water) with progressive increment in the size forming a thick layer at the periphery surrounding the core (due to an entanglement of polymeric chain) through which diffusion of the entrapped drug takes place (Sinha et al., 2011). The release of drug from the polymeric matrix could be determined by the thickness of the gelatinous layer, where diffusion of the drug further be governed by the viscosity of the swollen region, which in turn depends on the quantity of polymer in the matrix (Jian et al., 2012).

The swelling performance of 1% w/v gum katira of 5-FU loaded microsphere was depicted in the Figure 6. It can be observed that the incorporated gum was swelled to its limit at 10 h. During that period, 5-FU loaded microsphere shown a pattern of slow and continuous swelling followed by decreased in swelling percentage, which might be related to relaxation of the polymeric matrix, whereas, sodium alginate microspheres exhibited the faster swelling followed by immediately decrease in swelling as compared to gum katira microsphere (Kajjari et al., 2012). Thus, from the observations, it can be revealed that the swelling of incorporated polymer increases with the increase of gum/polymer content in the formulation, however, after a certain level of polymer concentration, the swelling decreases may be due to inhibition of water permeation within the matrix environment

*In vitro 5-FU release assay of optimized microsphere*

Optimized gum katira containing 5-FU microsphere were subjected to the in vitro drug release assay. The microsphere which targets the colon specifically should be stable at a wide range of pH during passing through the alimentary canal. The transition time for the stomach and small intestine are respectively 2h and 4h. The release of 5-FU from the final microspheres was carried out using a digital USP type II dissolution test apparatus (Lab India DS 8000 USP). The release from microsphere was evaluated in a simulating physiological condition (37°C, at pH 1.2 and pH 7.4). The in vitro release profiles of 5-FU were obtained by graphing the cumulative percentage of the drug released with respect to time. The experiment was performed over 24 h, and the results of 5-FU release from gum katira microsphere were expressed in Figure 6. Results demonstrated that there was a pronounced time prolongation on drug release. A biphasic release pattern of 5-FU was observed from the microsphere, where there was an initial release(it was due to the outer surface drug) followed by a sustained release from the core of the formulation. At first 2 h, the release of microsphere was approximately 16% of the entrapped drug, whereas cumulative release was found to be 83% of entrapped drug at 10 h, and finally, the release was recorded up to 96% at 24 h. 

*Evaluation of cytotoxicity assay*

Assay for cytotoxicity of the developed pharmaceutical dosage form represents a quick, easy, precise, safe and cheap assay to obtain the vital information on biological attributes of the molecule in a pharmaceutical on its basic tolerability. Therefore, these studies save resources and time, along with test animals and human liver in the process of testing a new molecule or novel formulation (Bácskay et al., 2017).In our current experiment, the cytotoxicity of free 5-FU, 5-FU loaded microsphere, and blank microsphere over HCT-116, MCF-7, and HeLa cell lines was assessed in 24 h. It had been observed that the both, free and microencapsulated 5-FU, inhibited viable cell, only after 24 h of incubation (Figure 7). In vitro MTT results showed that the blank microsphere exhibited no significant cytotoxicity after 24 h, indicating gum katira microsphere as a carrier of the drug without producing any toxic manifestation. It was consistent with the reports that gum katira is a natural carrier with no toxicity and biodegradable (as described earlier in toxicity study). Free drug and drug-loaded microsphere (from 0.5 to 10 µg/ml) similarly reduced viable cells, but there was a slight difference between free 5-FU and drug-loaded microsphere. The reason might be explained by the fact that the release of drug from the microsphere was incomplete (only approx 95% of the drug released from the microsphere up to 24 h in the invitro release study).

In conclusion, it can be concluded that natural gum katira was observed to be safe in rodents within the limit of our experimental condition. Further, 5-FU microsphere formulation can be formulated through optimization of processing parameters through multiple emulsion polymerization of the biocompatible gum katira. Expected outcomes of our in vitro findings indicated that the release of a remarkable quantity of greater than 83% within the time frame of 12 h from the entrapped drug of approximately 77%. Consistent release of the drug in colon pH could be effectively projected the release of the drug in the colonic environment for an adequate period to project the colon cancer. Simultaneously, targeted microsphere exhibited good cytotoxicity on HCT-116, MCF7, and HeLa cell lines, representing its efficacy comparable to the free drug. However, future research on stabilization will put importance on target-specific delivery of the entrapped therapeutic agent. In addition to fabrication, tailored microspheres need further evaluation of pharmacological, pharmacokinetic and toxicokinetic parameters to scale up the formulation technology for application in clinical research.
